# Analgesic Efficacy of Acupuncture on Chronic Pelvic Pain: A Systemic Review and Meta-Analysis Study

**DOI:** 10.3390/healthcare11060830

**Published:** 2023-03-11

**Authors:** Kent Yu-Hsien Lin, Yi-Chuan Chang, Wen-Chi Lu, Peddanna Kotha, Yi-Hung Chen, Cheng-Hao Tu

**Affiliations:** 1 Department of Gynecology, Ryde Hospital, Northern Sydney Local Health District, Sydney 2122, Australia; 2 Graduate Institute of Acupuncture Science, China Medical University, Taichung 404333, Taiwan; 3 Department of Chinese Medicine, China Medical University Beigang Hospital, Yunlin 651012, Taiwan; 4 International Master Program in Acupuncture, China Medical University, Taichung 404333, Taiwan; 5 Traditional Chinese Medicine Research Center, China Medical University, Taichung 404333, Taiwan; 6 Department of Photonics and Communication Engineering, Asia University, Taichung 41354, Taiwan

**Keywords:** chronic pelvic pain, pain management, acupuncture, monotherapy, adjunctive therapy

## Abstract

Chronic pelvic pain (CPP) is the pain occurred in the pelvic region longer than six months. The monotherapy of medicine may not adequate for the pain management of CPP and multidisciplinary approaches have been more recommended. The aim of this study is to evaluate the pain management efficacy of acupuncture compared with a control group on CPP. The articles of randomized controlled trial on CPP in PubMed and Embase databases were screened between January 2011 and September 2022 without language restriction to evaluate the treatment efficacy of acupuncture. The visual analogue scale/numerical rating scale (VAS/NRS) and total pain scores of National Institutes of Health—chronic prostatitis symptom index (NIH-CPSI) were served as outcome variables. Post-intervention mean scores were extracted and pooled for meta-analysis. Seventeen studies including 1455 patients were selected for meta-analysis. Both total pain scores of NIH-CPSI and VAS/NAS data revealed significant lower pain level in the acupuncture group than in the control group. Moreover, monotherapy with acupuncture revealed a significantly lower pain level than in the control group in both total pain scores of NIH-CPSI and VAS/NRS. These results indicated that acupuncture may have beneficial effects on pain management for CPP, even when administrated as a monotherapy.

## 1. Introduction

Chronic pelvic pain (CPP) is pain occurring in the pelvic region for longer than six months. CPP can manifest as cyclic or non-cyclic pain that may be associated with symptoms suggestive of lower urinary tract, sexual, bowel, myofascial, or gynecologic dysfunction [1]. The prevalence rate of CPP varies around the world. A previous study from the World Health Organization reported that the prevalence rates of non-cyclic CPP ranged from 2.1% to 24%, of cyclic CPP (associated with dysmenorrhea) ranged from 16.8% to 81%, and of intermitted CPP (associated with dyspareunia) ranged from 8% to 21.1% across different countries or regions [2]. Another study reported that the prevalence rate of CPP in females may be twice as high as in males [3]. A previous study using the United Kingdom primary care database reported that the annual prevalence of non-cyclic CPP in females may have been as frequent as migraine, back pain, and asthma between 1991 and 1995 [4]. It has been estimated that the direct annual medical cost (physician visit plus health care expenditure) and indirect economic lost (time lost from work) of CPP in 1994 were 2.8 billion and 555.3 million in the United States, respectively [5]. Thus, CPP is a common problem which may cause significant socioeconomic loses.

The management of CPP is challenging. A recent survey of the members of Royal College of Obstetricians and Gynaecologists reported that 45% responders considered the management of CPP in women in the United Kingdom is ‘poor’ or ‘very poor’ [6]. In addition, more than half (51%) of responders considered that ‘Pain management’ to be the most important aspect for the care of CPP in women, even above the ‘identification of cause of pain’. Non-surgical intervention for CPP often focus on pain relief if the cause of CPP is unknown [7]. Pharmacotherapy for the CPP may include prescript of analgesics, hormonal therapies, anticonvulsants, and/or antidepressants to relieve chronic pain and associated mood and sleep symptoms [8]. On the other hand, the non-pharmacotherapy also has been recommended to treat CPP, including physiotherapy, cognitive behavioral therapy, dietary therapy, neuromodulation, pain education, and lifestyle advice [9,10]. However, monotherapy may not be adequate for the management of CPP, and multidisciplinary approaches have been more recommended [9,10,11].

Acupuncture, an important intervention method in traditional Chinese medicine (TCM), is nowadays practiced worldwide for various diseases, especially in pain management. An individual patient data meta-analysis study with large sample size (with 17,922 patients) reported that acupuncture intervention has more analgesic effective than sham and no acupuncture intervention for back and neck pain, osteoarthritis, and chronic headache [12]. Similar results have been reported in a recent updated analysis (with 20,827 patients) for nonspecific musculoskeletal pain, osteoarthritis, chronic headache, or shoulder pain [13]. Furthermore, other meta-analysis studies have also reported superior efficacy of acupuncture intervention for the relief of postoperative pain [14], myofascial pain [15], cancer-related pain [16], as well as primary dysmenorrhea [17], endometriosis [18], irritable bowel syndrome [19], and chronic prostatitis/chronic pelvic pain syndrome (CP-CPPS) [20]. Since the revised standards for reporting interventions in clinical trials of acupuncture (STRICTA) has been introduced in 2010 to improve the quality of clinical trial report [21], in the present study we performed a systematic review on randomized controlled trial (RCT) of acupuncture for pain management of CPP from January 2011 to September 2022, and then conducted meta-analysis to determine the treatment efficacy of acupuncture in pain management of CPP, without prior registration of the review protocol on public website or database. Moreover, acupuncture treatment can be administered as monotherapy or as adjunctive therapy with other approaches. We also investigated the analgesic effect of acupuncture, whether administered as monotherapy or adjunctive therapy, to probe the possible difference between treatment strategies.

## 2. Materials and Methods

### 2.1. Eligibility Criteria

#### 2.1.1. Types of Studies

We included RCT that assessed pain experience to evaluate the treatment efficacy of acupuncture in CPP patients. Case report, case series, non-human RCT, review article, study protocol, and conference paper, abstract, and poster were excluded.

#### 2.1.2. Types of Participants

We included patients who suffer from CPP that may associated with endometriosis, pelvic gridle pain, idiopathic pelvic pain, inflammatory pelvic pain, and CP-CPPS. The acute pelvic pain may associate with primary dysmenorrhea, post-operative pain, constipation, and piriformis syndrome were excluded.

#### 2.1.3. Types of Interventions

The intervention based on acupoint stimulation, namely electroacupuncture (EA), manual acupuncture (MA), moxibustion, abdomen acupuncture, catgut implantation, laser acupuncture, and acupressure, were included in this study as acupuncture intervention. Rare types of acupuncture needle in clinical practice were excluded. Any other interventions, e.g., health education, standard care, western medication, physiotherapies, sham acupuncture and TCM medication, were recognized as control intervention in this study. The procedural intervention, e.g., nerve block injection, was excluded since it is hard to maintain a routine intervention.

#### 2.1.4. Outcome

The pain level was assessed with a validated scale or questionnaire, namely visual analog scale (VAS), numerical rating scale (NRS), and total pain scores of National Institutes of Health—chronic prostatitis symptom index (NIH-CPSI), were included in this study. Any study without pain assessment was excluded.

### 2.2. Data Sources and Study Selection

The RCT studies were searched for in the of PubMed and Embase database from 1 January 2011 to 30 September 2022 using the following search strategy: [(Endometriosis) or (pelvic pain) or (chronic pelvic pain) not (primary dysmenorrhea)] and [(Acupuncture) or (acupressure) or (electroacupuncture) or (meridians) or (moxibustion) or (needling)]. No language restriction was applied. Duplicated reports from PubMed and Embase were excluded by screening the title of reports. WC Lu and P Kotha independently selected the relevant reports based on the abstract. Then, Kent YH Lin and CH Tu reviewed the main text of reports and selected eligible studies for data extraction. Any disagreements were resolved by discussion among Kent YH Lin, CH Tu, and the arbiter YH Chen.

### 2.3. Data Extraction

Data was extracted from the included studies according to the predetermined data forms by WC Lu and then verified by YC Chang. The following items were extracted: publication information (journal, author, and year of publication); participants (sample size, sex, and age); acupuncture intervention (type of acupuncture, periods and frequency of intervention, and follow-up period if any); control (type of control, periods and frequency of intervention, and follow-up period if any); and pain assessment after intervention.

### 2.4. Risk of Bias Assessment

Kent YH Lin and CH Tu independently assessed the risk of bias for each included study using Risk of Bias tool 2.0 with the following 5 domains: bias arising from the randomization process; bias owing to departures from the intended interventions; bias from missing outcome data; bias in measurement of the outcome; and bias in selection of the reported result [22]. The overall risk and the risk of these 5 domains were judged with “Low”, “Some concerns”, or “High” level. Disagreements were resolved by discussion among two authors and the arbiter YH Chen.

### 2.5. Data Synthesis

The meta-analysis was performed using Review Manager software (RevMan v. 5.4.1). All VAS and NRS data were converted into scores between 0 and 10 by proportional method. To assess the effect of acupuncture on CPP, the data were analyzed using mean differences (MD) with 95% confidence interval (CI). The chi-square test was used to assess the heterogeneity of results among the included studies, and I^2^ scores were calculated to indicate the severity of the heterogeneity. The heterogeneity was considered significant if the p value of chi-square test was less than 0.05. A substantial heterogeneity among the studies was considered if I^2^ > 50%, whereas a serious heterogeneity was considered if I^2^ > 75%. When a serious heterogeneity was indicated, subgroup analysis was performed according to the type of acupuncture and type of control. A random effects model was used to synthesize the data. If only one appropriate study was allocated in a subgroup, descriptive synthesis of the findings was conducted. If the number of studies for pooling was more than 10, publication bias was assessed using a funnel plot.

## 3. Results

### 3.1. Study Selection

**Figure 1:**
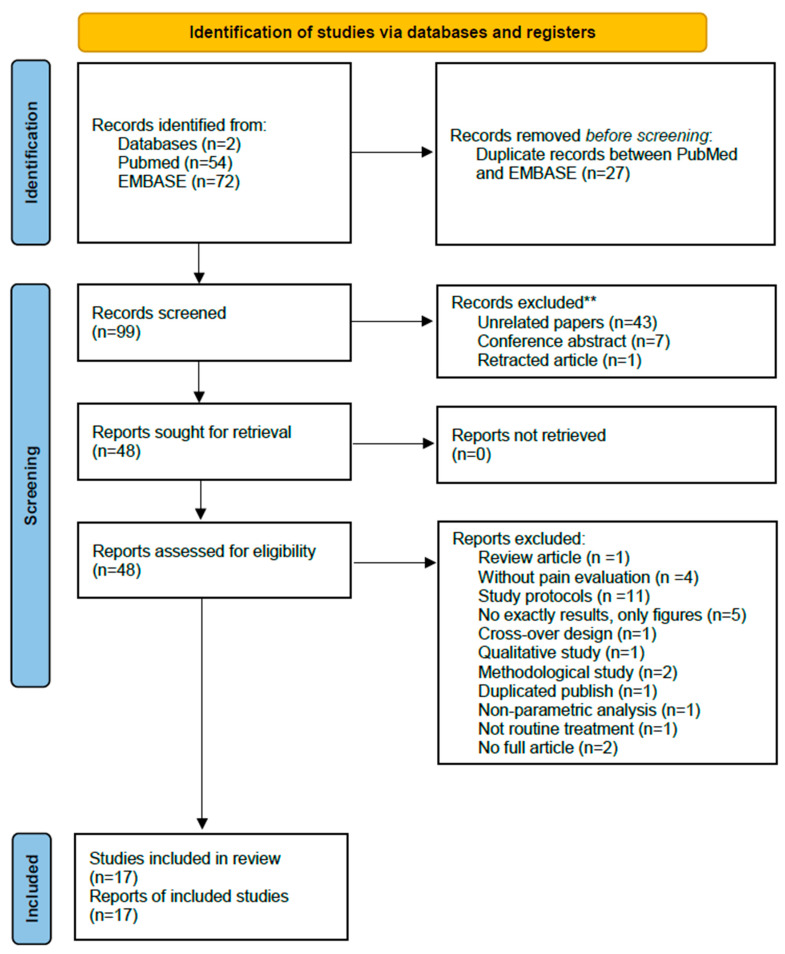
Flow diagram of the systemic review process.

A total of 126 reports were retrieved from PubMed and Embase. After removing the 27 duplicated reports, 99 reports were screened with their abstract, and 51 reports were excluded. The full texts of the remaining 48 reports were retrieved and reviewed. Thirty-one reports were excluded due to eligibility, and finally, there were 17 studies included for meta-analysis (Figure 1). All included studies were published between April 2011 and September 2022. Ten studies were published in Chinese [23,24,25,26,27,28,29,30,31,32] and 7 studies were published in English [33,34,35,36,37,38,39].

### 3.2. Study Characteristics

#### 3.2.1. Patients

A total of 1455 CPP patients (867 females and 588 males) were included into this meta-analysis. Among them, there were 728 CPP patients treated with acupuncture intervention, including 167 patients with EA (107 females and 60 males), 294 patients with MA (174 females and 120 males), 24 patients with moxibustion (24 females), 30 patients with abdomen acupuncture (30 females), 55 patients with ear acupuncture (55 females), 41 patients with laser acupuncture (41 females), and 117 patients with catgut implantation (117 males). On the other hand, there are 727 CPP patients received control intervention, including 157 patients with standard care (157 females), 333 patients with western medication (122 females and 211 males), 72 patients with TCM medication (72 females), 85 patients with physiotherapy (85 females), and 80 patients with sham acupuncture (80 males) (Table 1).

The age range of CPP patients was 18 to 50 years. The possible etiology of CPP were idiopathic chronic pelvic pain, inflammatory pelvic pain, endometriosis, pregnancy-related pelvic gridle pain, and category IIIB of CP-CPPS (Table 1).

#### 3.2.2. Acupuncture Interventions

Among the 17 included RCTs, EA was conducted as acupuncture intervention in 5 RCTs. The other 6 RCTs conducted with MA as their acupuncture intervention, and 2 RCTs conducted with catgut implantation as their acupuncture intervention. Moxibustion, abdomen acupuncture, ear acupuncture, and laser acupuncture served as acupuncture intervention in the remained 4 RTCs, respectively (Table 2). The acupuncture intervention was administrated as an adjunctive therapy in 6 RCTs, whereas acupuncture intervention was administrated as a monotherapy in 11 RCTs (Table 2). The intervention periods were ranged from 2 weeks to 6 months (Table 2).

#### 3.2.3. Controls

Different types of control have been conducted in these 17 RCTs. Western medication has been used as a control intervention in 8 RCTs, whereas TCM medication served as a control intervention in 3 RCTs. Standard care, sham acupuncture, and physiotherapies were served as control interventions in 2 RCTs, respectively (Table 2). The intervention periods for control intervention were ranged from 2 weeks to 6 months (Table 2).

#### 3.2.4. Outcome Measures

The pain level was assessed with VAS in 8 RCTs, with NRS in 2 RCTs, and with total pain scores of NIH-CPSI in 7 RCTs (Table 1). In addition to the assessment of pain level after intervention, 5 RCTs assessed pain level with additional follow-up period ranging from 12 weeks to one year (Table 1).

### 3.3. Risk of Bias

**Figure 2:**
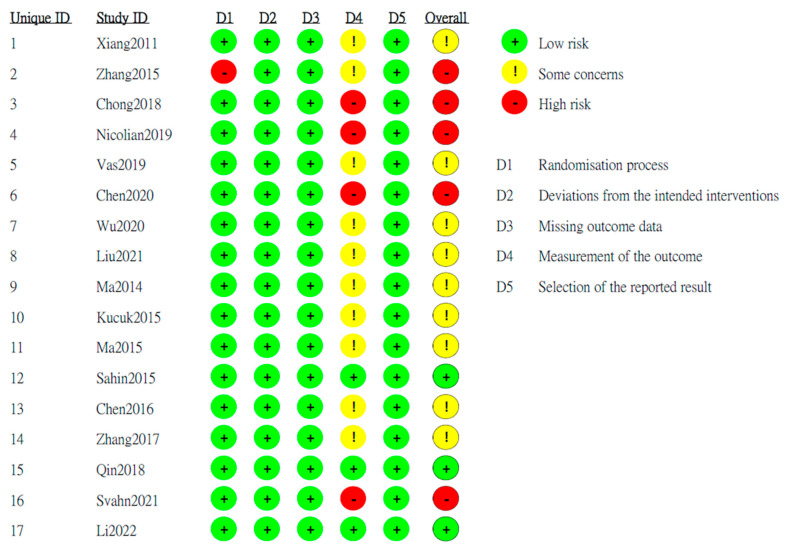
Summary for the items of risk of bias in each included study [23,24,25,26,27,28,29,30,31,32,33,34,35,36,37,38,39].

Among these 17 RCTs, 14 RCTs were judged at low risk of bias in the randomization process but one RCT was judged at high risk of bias in the randomization process. All 17 RCTs were judged at low risk of bias in deviation from the intended interventions, missing outcome data, and selection of the reported result. However, 12 RCTs were raised with some concerns of the risk of bias in measurement of the outcome, whereas 3 RCTs were judged at low risk of bias in measurement of the outcome. Taken together with these judgements of risk from 5 domains, there were 3 RCTs have low overall risk of bias, 11 RCTs have some concerns of the overall risk of bias, and 1 RCT have high overall risk of bias (Figure 2).

### 3.4. Pain Management

#### 3.4.1. Overall Effect

**Figure 3:**
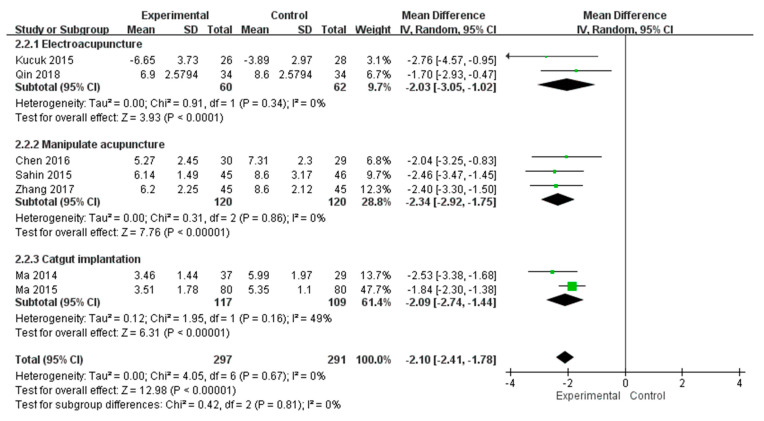
Meta-analysis of the studies evaluating the pain management effect of acupuncture using total pain scores of NIH-CPSI [23,27,28,32,34,36,37].

**Figure 4:**
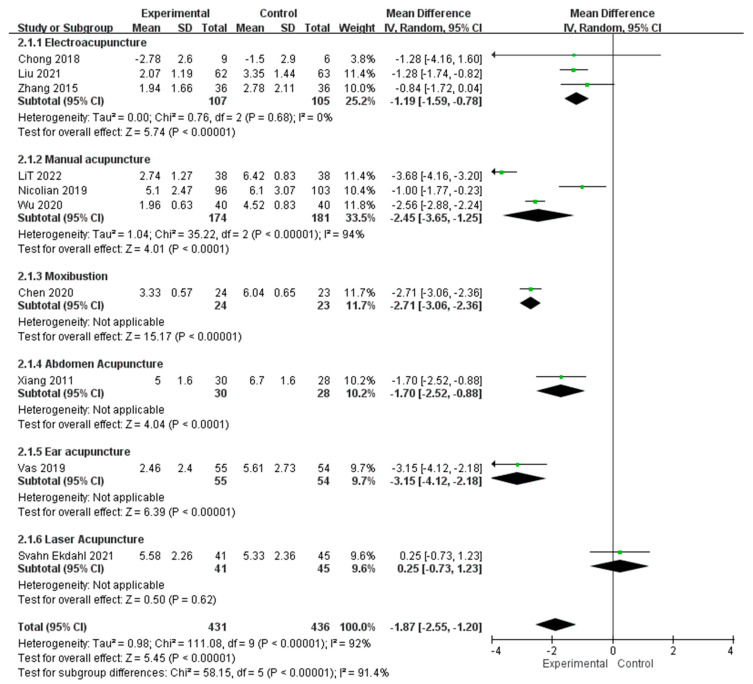
Meta-analysis of the studies evaluating the pain management effect of acupuncture using VAS/NRS and subgroup analysis with different acupuncture interventions [24,25,26,29,30,31,33,35,38,39].

Since the total pain scores of NIH-CPSI also evaluate the frequency and the region of pain, which are not evaluate by the single-dimensional VAS/NRS, the data has been separated and pooled according to the types of outcome measurement (i.e., NIH-CPSI or VAS/NRS) for meta-analysis. The pain assessed with total pain scores of NIH-CPSI post-intervention data pooled from 7 RCTs revealed a significantly lower pain level in acupuncture than in control (MD = −2.10, 95% CI [−2.41, −1.78], *p* < 0.00001). No significant heterogeneity has been found among these 7 RCTs (Chi-square = 4.05, *p* = 0.67; I^2^ = 0%) (Figure 3). The post-intervention data pooled from 10 RCTs which assessed pain level with VAS or NRS also revealed a significantly lower pain level with acupuncture intervention than with control intervention (MD = −1.87, 95% CI [−2.55, −1.20], *p* < 0.00001). However, a serious heterogeneity has been found among these 10 RCTs (Chi-square = 111.08, *p* < 0.00001; I^2^ = 92%) (Figure 4).

#### 3.4.2. Subgroup Analysis of Acupuncture Interventions

Considering that serious heterogeneity has been found among the RCTs that assessed pain level with VAS or NRS, we conducted a subgroup analysis according to the types of acupuncture intervention. Pooled post-intervention data from 3 RCTs with EA intervention revealed significantly lower pain level in acupuncture than in control (MD = −1.19, 95% CI [−1.59, −0.78], *p* < 0.00001). Pooled post-intervention data from 3 RCTs with MA intervention also revealed significantly lower pain level in acupuncture than in control (MD = −2.45, 95% CI [−3.65, −1.25], *p* = < 0.0001). For moxibustion, a single RCT revealed significant lower pain level in acupuncture than in control (MD = −2.71, 95% CI [−3.06, −2.36], *p* < 0.00001). Another RCT with abdomen acupuncture revealed significantly lower pain level in acupuncture than in control (MD = −1.70, 95% CI [−2.52, −0.88], *p* < 0.0001). One RCT with ear acupuncture revealed significant lower pain level in acupuncture than in control (MD = −3.15, 95% CI [−4,12 −2.18], *p* < 0.00001). However, no significant difference in pain level between acupuncture and control was found in an RCT with laser acupuncture as acupuncture intervention (MD = 0.25, 95% CI [−0.73, 1.23], *p* = 0.62). Among these 6 different types of acupuncture intervention, significant subgroup differences have been found (Chi-square = 58.15, *p* < 0.00001; I^2^ = 91.4%) (Figure 4).

#### 3.4.3. Subgroup Analysis of Control Interventions

**Figure 5:**
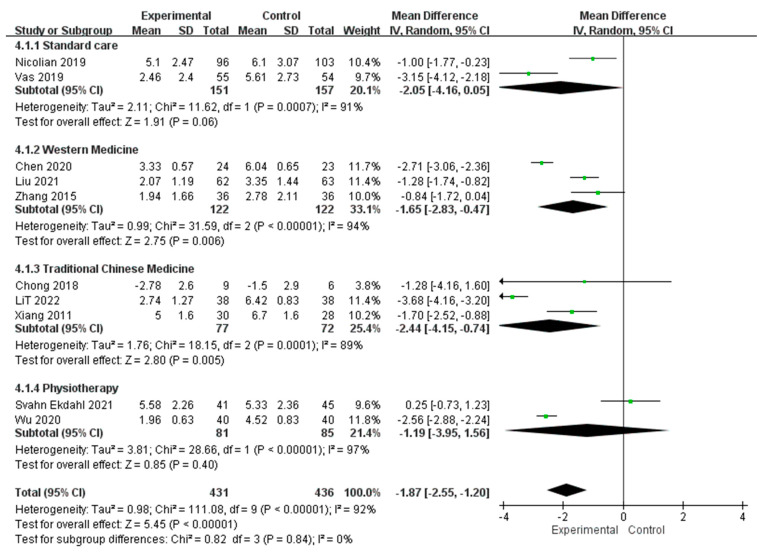
Subgroup analysis of the studies evaluating the pain management effect of acupuncture using VAS/NRS with different control interventions [24,25,26,29,30,31,33,35,38,39].

To test whether the serious heterogeneity among RCTs that assessed pain level with VAS or NRS may be contributed from different types of control intervention, we conducted a subgroup analysis according to the types of control intervention. Pooled post-intervention data from 2 RCTs with standard care revealed no significant change in pain level between acupuncture and control (MD = −2.05, 95% CI [−4.16, 0.05], *p* = 0.06). Pooled post-intervention data from 3 RCTs with Western medication revealed significantly lower pain level in acupuncture than in control (MD = −1.65, 95% CI [−2.83, −0.47], *p* = 0.006). Pooled post-intervention data from other 3 RCTs with traditional Chinese medication revealed significantly lower pain level in acupuncture than in control (MD = −2.44, 95% CI [−4.15, −0.74], *p* = 0.005). For 2 RCTs with physiotherapy as the control intervention, no significant difference was found between acupuncture and control (MD = −1.19, 95% CI [−3.95, 1.56], *p* = 0.40). Among these 4 different types of control intervention, no significant subgroup differences were found (Chi-square = 0.82, *p* = 0.84; I^2^ = 0%) (Figure 5).

#### 3.4.4. Subgroup Analysis of Therapeutic Strategy

**Figure 6:**
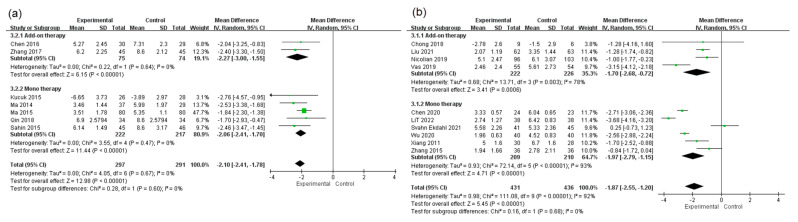
Subgroup analysis of therapeutic strategy on (**a**) total pain scores of NIH-CPSI [23,27,28,32,34,36,37] and (**b**) VAS/NRS [24,25,26,29,30,31,33,35,38,39].

Considering that a multidisciplinary approach for pain management has been more recommended for CPP patients [9,10], we conducted a subgroup analysis for different therapeutic strategy (adjunctive therapy or monotherapy). For the RCTs which assessed pain level with total pain scores of NIH-CPSI, pooled post-intervention data from 2 RCTs with adjunctive acupuncture intervention revealed significantly lower pain level in acupuncture than in control (MD = −2.27, 95% CI [−3.00, −1.55], *p* < 0.00001). Pooled post-intervention data from 5 RCTs with monotherapy of acupuncture also revealed significantly lower pain level in acupuncture than in control (MD = −2.06, 95% CI [−2.41, −1.70], *p* < 0.00001). No significant subgroup differences were found (Chi-square = 0.28, *p* = 0.60; I^2^ = 0%) (Figure 6a). For the RCTs which assessed pain level with VAS or NRS, pooled post-intervention data from 4 RCTs with adjunctive acupuncture intervention revealed significant lower pain levels in acupuncture than in control (MD = −1.70, 95% CI [−2.68, −0.72], *p* = 0.0006). Pooled post-intervention data from 4 RCTs with monotherapy of acupuncture also revealed significantly lower pain levels in acupuncture than in control (MD = −1.97, 95% CI [−2.79, −1.15], *p* < 0.00001). No significant subgroup differences were found (Chi-square = 0.17, *p* = 0.68; I^2^ = 0%) (Figure 6b).

#### 3.4.5. Publication Bias

**Figure 7:**
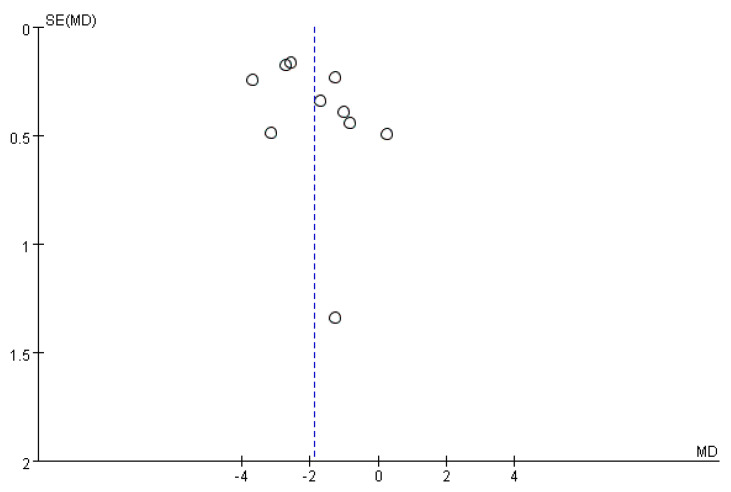
The funnel plot with the studies using VAS/NRS as measurement variable.

For the pooling studies that used VAS or NRS scores, no possible publication bias was identified using the funnel plot (Figure 7). Since the number of pooling studies that used NIH-CPSI scores were less than 10, no funnel plot asymmetry was investigated.

## 4. Discussion

In the present study, we conducted a systematic review of RCTs from PubMed and Embase to evaluate the treatment efficacy of acupuncture comparing to other interventions for CPP. The meta-analysis on post-intervention data revealed that acupuncture intervention has less pain level than control intervention for CPP, as measured by both VAS/NRS and total pain scores of NIH-CPSI assessment. Furthermore, our analysis of both adjunctive therapy and monotherapy revealed that acupuncture intervention has lower pain levels than control intervention for CPP. These results indicated that acupuncture may have beneficial effects for pain management of CPP, even when administrated as a monotherapy.

Although different pain assessment methods have been conducted among these 17 RCTs, acupuncture intervention has shown better treatment efficacy than control intervention in pain management of CPP. Since almost all acupuncture methods (expect laser acupuncture) have revealed better treatment efficacy than control intervention on CPP, the analgesic effect of acupuncture might be underpinned by multiple mechanisms at different levels of nervous system. Previous studies have indicated that acupuncture analgesia may be associated with neural activity, neurotransmitters, and cytokines in the peripheral, central and autonomic nervous systems [40,41,42]. Although the possible mechanisms between different acupuncture interventions and the possible correlations represented among these RCTs cannot be probed in the present study, it seems that the analgesic effect of acupuncture intervention on CPP may be more contributed from the central mechanisms than the peripheral mechanisms. This notion is supported by the fact that more remote but not local acupoints were selected for acupuncture treatment in these RCTs. In TCM, it is well known that different acupoints may have different effects on the body. These acupoints have been noted for their curative effects that can produce a certain clinical effect regardless of the intervention method applied. Hence, more studies are needed to clarify the possible contributions from central and peripheral mechanisms to optimize the treatment effect of acupuncture.

Moreover, the pain from CPP can be much relieved by acupuncture with monotherapy compared to control interventions. Acupuncture treatment is flexible and widely used, and pain control is one of the main indications of acupuncture treatment, especially for chronic pain [13]. In the present study, the clinical pain management efficacy of acupuncture, regardless of the intervention method, was better than that of Western medication and TCM medication, and have trans of better efficacy than standard care. It has been suggested that combining different approaches may be one of the more effective treatment strategies for CPP [9,10,11]. Our results further reveal that acupuncture can relieve the pain of CPP whether used in adjunctive therapy or monotherapy. In clinical practice, acupuncture has the characteristics of being able to cooperate with other treatment methods, and different acupuncture intervention process and methods can also be combined with each other in most situations. Acupuncture treatment is characterized by low cost and few side effects [35,43]. Combined with the results of the present study, acupuncture may not only beneficial for pain management on CPP even conducted with the strategy of monotherapy, but also may lower the direct or indirect huge medical resource consuming and/or personal economic costs.

There are many causes of CPP, including anatomical, functional, degenerative, organic and other different causes [1]. The results of the present study revealed that acupuncture-related treatments can generally alleviate the pain of CPP with different etiologies. Although acupuncture approach is generally effective in the treatment of CPP, there are very few published literatures on specific acupuncture methods. In the future, a focus on specific acupuncture methods for various causes of CPP could be explored. Large-scale or multi-center RCTs can be considered to further verify the clinical efficacy of acupuncture for pain management in CPP. These results may help to gain a better understanding of the underlying mechanisms of acupuncture treatment on CPP and verify the cost-effectiveness between acupuncture compared to other interventions.

Some limitations of the present study should be noted. First, there is only one study indexed for some interventional methods such as moxibustion, ear acupuncture, abdomen acupuncture, and laser acupuncture, respectively. More studies are needed to verify the results from these RCTs. Second, pooling a few studies may lead to difficulties in obtaining a precise estimate of the between-studies variance [44]. Thus, a random effects model was implemented in the present study. Third, although the revised STRICTA guideline has been purposed in 2010, the majority of the included RCTs in the present study still have some concerns regarding the risk of bias, especially in the domain of “measurement of the outcome”. This is mainly due to the nature of self-reported subjective pain intensity (patients assessed the outcome themselves). Hence, the objective pain assessment system (e.g., automatic facial expression system) should be introduced in future studies.

## 5. Conclusions

Acupuncture intervention effectively help CPP patients on their pain management. The various methods of acupuncture, e.g., MA, EA, catgut implantation, moxibustion, abdominal acupuncture, and ear acupuncture, have higher treatment efficacy than Western medication or TCM medication. Moreover, the monotherapy of acupuncture has a higher efficacy for pain management than control interventions for CPP, as well as recommended adjunctive therapy. These results indicated that acupuncture may have beneficial effects for pain management of CPP, even when administrated as a monotherapy. Acupuncture may be potentially advantageous for patients who are intolerant to drugs, experience refractory pain, or have comorbidities, and it may alleviate the psychological symptoms.

## Figures and Tables

**Table 1:** Main characteristics of included studies.

Studies	Population Type	Sample Size Intervention/Control	Age (Mean) Intervention/Control	Outcomes	Course of Treatment	Follow-Up Visit
Xiang 2011 [30]	Patients with endometriosis	30/28	34.6/34.6	VAS ^4^ (0–10) McGill pain questionnaire	3 months (3 MC ^7^ cycle)	NA ^8^
Zhang 2015 [31]	Patients with endometriosis	36/36	33/35	VAS ^4^ (0–10)	6 months	NA ^8^
Chong 2018 [33]	Women with CPP ^1^ over 6 months	9/6	34.7/31.4	NRS ^5^ (0–10)	4 weeks	12th week
Nicolian 2019 [35]	Pregnant women with pelvic girdle and low back pain	96/103	31/30.7	NRS ^5^ (0–10)	4 weeks	NA ^8^
Vas 2019 [39]	Pregnant women at 24–36 weeks of gestation with pregnancy-related lower back and/or posterior pelvic girdle pain	55/54	31.4/31.7	VAS ^4^ (0–100)	2 weeks	3 months postpartum, and 1 year after randomization
Chen 2020 [24]	Patients with ovarian endometriosis dysmenorrhea	24/23	33/32	VAS ^4^ (0–10)	3 months	NA ^8^
Wu 2020 [29]	Patients with postpartum pelvic girdle pain	40/40	28/28	VAS ^4^ (0–10)	3 weeks	NA ^8^
Liu 2021 [26]	CPP ^1^ patients with sequelae of pelvic inflammatory disease	62/63	35/36	VAS ^4^ (0–10) Lower abdomen	3 months (3 MC cycles)	NA ^8^
Svahn Ekdahl 2021 [38]	Patients with pelvic girdle pain 12–48 weeks	41/45	30.5/31.1	VAS ^4^ (0–10)	5 weeks	NA ^8^
Li 2022 [25]	Endometriosis related pelvic pain of cold coagulation and blood stasis.	38/38	33/37	VAS ^4^ (0–10)	4 weeks	3-month after treatment
Ma 2014 [28]	Patients with CP/CPPS ^2^ (NIH ^3^ category IIIB)	37/29	31/33	NIH-CPSI ^6^ score Pain (0–21)	8 weeks	NA ^8^
Kucuk 2015 [34]	Patients with CP/CPPS ^2^	26/28	All subjects’ mean: 33.3	NIH-CPSI ^6^ pain total score (0–21)	7 weeks	NA ^8^
Ma 2015 [27]	Patients with CP/CPPS ^2^ (NIH ^3^ category IIIB)	80/80	35/33	NIH-CPSI ^6^ score Pain (0–21)	8 weeks	NA ^8^
Sahin 2015 [37]	Patients with CP/CPPS ^2^ (NIH ^3^ category IIIB)	45/46	32.1/32.8	NIH-CPSI ^6^ pain score (0–21)	6 weeks	At 8th, 16th, and 24th week
Chen 2016 [23]	Patients with CP/CPPS ^2^	30/29	34/34	NIH-CPSI ^6^ Pain total score (0–21)	24 days	NA ^8^
Zhang 2017 [32]	Patients with CP/CPPS ^2^ (NIH ^3^ category IIIB)	45/45	All subjects’ mean: 30.6	NIH-CPSI ^6^ Pain total score (0–21)	4 weeks	NA ^8^
Qin 2018 [36]	Patients with CP/CPPS	34/34	33.8/35.1	NIH-CPSI ^6^ Pain total score (0–21)	8 weeks	32 weeks

^1^ CPP: Chronic pelvic pain; ^2^ CP/CPPS: Chronic prostatitis/chronic pelvic pain syndrome; ^3^ NIH: National institute of health; ^4^ VAS: Visual analog scale; ^5^ NRS: Numerical rating scale; ^6^ NIH-CPSI: National Institutes of Health—Chronic Prostatitis Symptom Index; ^7^ MC: Menstrual cycle; ^8^ NA: Not available.

**Table 2:** Intervention details of included studies.

Studies	Intervention		Treatment Schedule
	Acupuncture Group	Control Group	
Xiang 2011 [30]	Abdominal acupuncture Acupoint: CV12, CV10, CV6…etc.	Chinese Medicine: Tianqi Tongjing Capsule	For 3 MC cycle, once each 1~2 days, at least 3 time each MC cycle
Zhang 2015 [31]	Electro-acupuncture Acupoints: CV6, CV4, CV3, EXCA1, SP8, SP6, LI4, LR3. EA on CV4 and CV3	Mifeprisone tablet 12.5 mg once a day for 6 months	once every 2 days for 6 months, suspend treatment during menstruation
Chong 2018 [33]	BMEA treatment (BMEA + TCM HC) Acupoints: the most painful Ashi points (tight and tender points) with electrical stimulation	TCM HC interventions twice a week for 4 weeks	Twice a week for 4 weeks
Nicolian 2019 [35]	Acupuncture plus standard care Acupoints: Bilateral BL40 and Ashi points	Standard care: pregnancy belt, lifestyle recommendations, and exercises	Twice in first week, then once per week in next 3 weeks. 5 sessions.
Vas 2019 [39]	Standard obstetric care plus ear acupuncture	Standard obstetric care	2 weeks
Chen 2020 [24]	Herb-separated moxibustion was applied at hypogastrium and lumbosacral area for 30 min	Giving ibuprofen sustained-release capsule when necessary	Once a week for 3 months
Wu 2020 [29]	Acupuncture plus manipulative reduction Acupoints: GV4, BL25, GV3, BL32, BL33, GB30, and GB34	Manipulative reduction	once every two days, three times a week and 3 treatments taken as one course. In total, 3 courses of treatment were required (3 weeks)
Liu 2021 [26]	Electro-acupuncture Acupoints: CV4, ST28, ST29, BL23, and BL32 Disperse-dense wave, 2 Hz/15 Hz of frequency for 30 min	Ibuprofen sustained-release capsules 10 days before menstruation, 0.3 g each time, once a day per menstrual cycle for 3 menstrual cycles	once a day, 10 days per menstrual cycle, for 3 menstrual cycles
Svahn Ekdahl 2021 [38]	Manual acupuncture Acupoint: GV 20, bil LI 4, bil BL 26, bil BL 32, bil BL 33, bil BL 54, bil KI 11, bil BL 60, bil EX 21, bil GB 30, bil SP 12, bil ST 36	TENS device at home. Treatment area: unilaterally or bilaterally over the sacroiliac joint and gluteal muscles for dorsal pelvic pain or in the groin area for pubic pain. Intensity: High frequency stimulation (80 Hz)	Acupuncture: 10 acupuncture sessions (two sessions per week) TENS: At least 30 min per day for 5 weeks.
Li 2022 [25]	CO2 laser acupuncture (100 mW, area 2 cm, distance to skin 3 cm) Acupoint: bil Zigong (EX-CA 1)	Sham laser acupuncture (0 mW, area 2 cm, distance to skin 3 cm) Acupoint: bil Zigong (EX-CA 1)	Once every other day, 30 min each time, 3 times a week for 4 weeks.
Ma 2014 [28]	Catgut embedding acupuncture Acupoints: SP6, CV1, LI11, ST36, CV3, and BL23	Tamsulosin hydrochloride, 0.2 mg, once daily Indomethacin, 75 mg, once daily	Once each 2-week for 8 weeks
Kucuk 2015 [34]	Electro-acupuncture Acupoints: UB28, GB41, LIV3, LI4, SP6, and SP8	Levofloxacin 500 mg once daily and ibuprofen 200 mg twice daily for 6 weeks	Twice a week for 7 weeks
Ma 2015 [27]	Catgut embedding acupuncture Acupoints: SP 6, CV 1, CV 2, BL 54, and BL 23	Tamsulosin hydrochloride 0.2 mg once daily Indomethacin 75 mg three times daily	Once every two weeks; the treatment of 4 weeks made one session, and two sessions were required.
Sahin 2015 [37]	Acupuncture Acupoints: Bilateral BL33, BL34, BL54, CV1, CV4, SP6, and SP9	Sham acupuncture Acupoints: 1 cm left of each selected acupoint bilaterally (BL33, BL34, BL54, CV1, CV4, SP6 and SP9)	The acupuncture needles were lasted for 20 min in both groups and half of this period covered by needle stimulation through rotation. The procedure was repeated every week for a period 6 week
Chen 2016 [23]	Manual acupuncture plus western medicine Scalp points GV24, GV22, GV21, GV20, BL6, BL7. Body points: CV3, CV4, BL28, and BL32	Levofloxacin 200 mg twice daily Tamsulosin hydrochloride 0.2 mg once daily	Once a day, 12 days treatments as one session and total 2 session were given.
Zhang 2017 [32]	Triple acupuncture at the Qugu (RN2) acupoint 20 min, twice a week plus Levofloxacin Mesylate Tablets 0.5 g three times daily, and Terazosin Hydrochloride Capsules 2 mg once daily for 4 weeks	Levofloxacin Mesylate Tablets 0.5 g three times daily, and Terazosin Hydrochloride Capsules 2 mg once daily for 4 weeks	Twice a week for 4 weeks
Qin 2018 [36]	Manual acupuncture Acupoints: Bilateral BL33, BL23, SP6, and BL35	Sham non-penetration acupuncture Same procedure as Verum acupuncture groups with placebo needles	3 treatment sessions per week for 8 consecutive weeks for a total of 24 sessions

CV: Conception Vessel; SP: Spleen Meridian; LI: Large Intestine Meridian; BL: Bladder Meridian; GV: Governor Vessel; GB: Gallbladder Meridian; ST: Stomach Meridian; KI: Kidney Meridian; EX: Ex-Meridian; UB: Urinary Bladder Meridian; RN2: Qugu; EA: Electropuncture; TENS: Transcutaneous electrical nerve stimulator; BEMA: The meridian balance method electroacupuncture; TCM HC: Traditional Chinese medicine health consultation; bil: bilateral.

## Data Availability

The data presented in this study are openly available in PubMed and Embase.
